# Pilot Program of Newborn Screening for 5q Spinal Muscular Atrophy in the Russian Federation

**DOI:** 10.3390/ijns9020029

**Published:** 2023-05-16

**Authors:** Kristina Mikhalchuk, Olga Shchagina, Alena Chukhrova, Viktoria Zabnenkova, Polina Chausova, Nina Ryadninskaya, Dmitry Vlodavets, Sergei I. Kutsev, Alexander Polyakov

**Affiliations:** 1 Research Centre for Medical Genetics, Moskvorechye St., 1, 115522 Moscow, Russia; 2 Russian Children Neuromuscular Center, Veltischev Clinical Pediatric Research Institute, Pirogov Russian National Research Medical University, Taldomskaya Str. 2, 125412 Moscow, Russia

**Keywords:** SMA, *SMN1*, newborn, screening, pilot, the Russian Federation

## Abstract

5q spinal muscular atrophy (5q SMA) is one of the most common autosomal recessive disorders in the Russian Federation. The first medication to treat 5q SMA was registered in the Russian Federation for treatment of all 5q SMA types in 2019, and the last of the three currently available in December 2021. We launched the pilot newborn screening (NBS) program for 5q SMA in Moscow, the Russian Federation, starting in 2019. During the pilot program, 23,405 neonates were tested for the deletion of exon 7 of the *SMN1* gene, the most common cause of 5q SMA. We used the SALSA^®^ MC002 SMA Newborn Screen Kit (MRC Holland) to specifically detect homozygous deletions of *SMN1* exon 7. We used the restriction fragment length polymorphism (RFLP) approach to validate detected homozygous deletions and the SALSA MLPA Probemix P060 SMA Carrier Kit (MRC Holland) to determine the *SMN2* exon 7 copy number to prescribe gene therapy for 5q SMA. Three newborns with a homozygous deletion of the *SMN1* gene were detected. The calculated birth prevalence of 1:7801 appears to be similar to the results in other European countries. The children did not show any signs of respiratory involvement or bulbar weakness immediately after birth. Until now, no 5q SMA case missed by NBS has been detected.

## 1. Introduction

Hereditary spinal muscular atrophy (SMA) is a large, genetically heterogeneous group of disorders characterized by progressive degeneration and death of motor neurons in the anterior horns of the spinal cord and, in some cases, in the nuclei of the brain stem [1]. 5q SMA is one of the most common autosomal recessive disorders in the world and the most frequent cause of genetically related infant mortality [2]. In Russia, the carrier frequency is 1/36, and the estimated disease frequency is 1:5184 newborns [3,4,5].

The cause of 5q SMA is mutations in the *SMN1* gene in a homozygous or compound heterozygous state. The *SMN1* gene is located in an inverted repeat region on the 5q chromosome; the repeat also includes the *SMN2* gene, which has minimal differences from *SMN1*—the coding sequence of *SMN2* differs from the sequence of *SMN1* by one nucleotide (c.840C>T) and leads to alternative splicing of exon 7. As a result, only 10% of normal SMN protein is produced [6,7,8,9]. In 95–97% of patients with 5q SMA, the cause of the disease is the deletion of exon 7 of the *SMN1* gene in a homozygous state.

Currently, three medications to treat 5q SMA are registered in Russia. The first medication, an antisense oligonucleotide, Nusinersen (Spinraza), was registered in the Russian Federation for treatment of all 5q SMA types in 2019. The second medication, Risdiplam (Evrysdi), a small molecule modifying splicing, was registered in Russia in December 2020. A novel gene therapy for the treatment of 5q SMA (the third medication), Onasemnogene abeparvovec (Zolgensma), received a registration certificate in December 2021. Thus, all three of the currently available medications for 5q SMA treatment are registered in the Russian Federation. This fact creates conditions for successful treatment and screening implementation in the country. Presymptomatic treatment is proven to decrease lethality and disability rates most effectively [10]. Considering the age of disease manifestation, it is reasonable to detect newborns with homozygous deletions during neonatal screening. There is no biochemical marker for 5q SMA; therefore, the only possible screening approach is molecular genetic diagnostics aimed at detecting the deletion of exon 7 of the *SMN1* gene in a homozygous state.

Since 2006, neonatal screening for the following five hereditary disorders—phenylketonuria, cystic fibrosis, galactosemia, congenital adrenal hyperplasia, and congenital hypothyroidism—has been carried out in Russia. A pilot project of newborn screening for 5q SMA has been started in Moscow to estimate the incidence of the disease and identify newborns with 5q SMA who can be given therapy at the presymptomatic stage of the disease.

The aim of this pilot project was: (1) to refine methodological approaches for neonatal screening for 5q SMA at the pre-mass neonatal screening stage in the Russian Federation; (2) to determine the sensitivity, specificity, and feasibility of the SALSA^®^ MC002 SMA Newborn Screen Kit (MRC Holland) without DNA isolation; and (3) to directly measure the frequency of 5q SMA due to homozygous exon 7 deletion of the *SMN1* gene in a multinational mixed population sample from three maternity wards in Moscow.

## 2. Materials and Methods

The following maternity wards participated in the program: the maternity wards of S. S. Yudin City Clinical Hospital and E. O. Mukhin City Clinical Hospital, and clinical hospital No. 2 of the I. M. Sechenov First Moscow State Medical University. All mothers of 23,405 newborns signed informed consent to collect samples for the pilot 5q SMA screening project, and nobody declined participation in the study. All parents were informed that at the time of the start of screening, only 2 drugs for the treatment of 5q SMA were registered in the Russian Federation. Samples were taken from all newborns during the pilot project from August 2019 to January 2022.

Newborn screening for the 5 diseases in the Russian Federation was performed on the fourth day of the newborn’s life and then sent to reference laboratories for screening. Dried blood spots on Whatman^®^ 903 cards for neonatal screening for 5q SMA were taken together with samples for screening for the 5 diseases and sent immediately to the Research Center for Medical Genetics for further diagnosis. Samples of newborns were collected and examined separately from samples of patients with suspected 5q SMA admitted for routine diagnosis. The DBS were transferred every day to the Research Center for Medical Genetics on days 4–6 of life. The 5q SMA screening results were available on days 6–8 of life.

**Figure 1:**
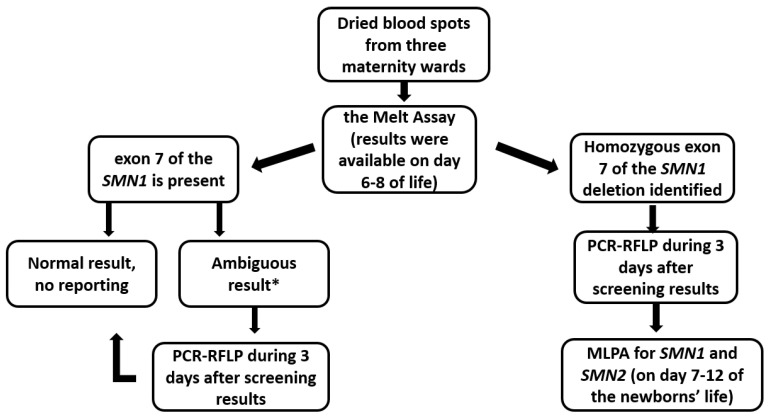
Algorithm used in the pilot program of newborn screening for 5q SMA in Moscow. * “Ambiguous result” means the intensity of the signal of exon 7 of the *SMN1* gene in such samples was significantly (5–9 times) lower than the intensity of the signal of exon 7 of the *SMN2* gene, while during the analysis of other samples the intensity ratio did not exceed 1:2–1:3.

The algorithm for screening and follow-up is shown in Figure 1. The pilot project was carried out via the melt assay using the SALSA^®^ MC002 SMA Newborn Screen Kit (MRC Holland) according to the manufacturer’s protocol. The assay can be performed on a crude DNA extract prepared from a 1.5 mm or 3.2 mm punch of a DBS card. After screening directly from DBS cards, DNA extraction from positive and ambiguous DBS samples was carried out for PCR-RFLP to confirm the presence of the homozygous deletion of exons 7 and 8 of the *SMN1* gene and to make a diagnosis of 5q SMA; the analysis took up to 3 days, according to the laboratory’s timing. The positive samples were validated using the restriction fragment length polymorphism (RFLP) (exon 7—5′AAAGCTATCTATATATAGCTATCG*AT, 3′TCACTTTCATAATGCTGGCAGAC; exon 8—5′GTAATAACCAAATGCAATGTGAA, 3′CTACAACACCCTTCTCACAGG; * nucleotide, implemented into the primary sequence to create a restriction site for BseJ1 endonuclease (Thermo Scientific, Vilnius, Lithuania). Details on the performance of the PCR-RFLP analysis during the pilot project are provided in Appendix A. Immediately after receiving the screening result, the parents were contacted to summon them to the Research Center for Medical Genetics for blood sampling of the whole family (referring to parents and siblings). Confirmatory molecular tests were performed on a fresh blood sample with signed informed consent; this strategy was chosen also to rule out sampling mismatch at the maternity ward since we did not expect false positive results. DNA extraction from peripheral blood leukocytes of positive samples was performed for direct automated Sanger sequencing and MLPA. DNA extraction from peripheral blood leukocytes and DBS to conduct the following tests was performed using the Wizard^®^ Genomic DNA Purification Kit (Promega, Madison, WI, USA) according to the manufacturer’s protocol. MLPA was a reference method to confirm the presence of homozygous deletion of exons 7 and 8 of the *SMN1* gene as well as establish the *SMN2* copy number for subsequent gene therapy for the newborn and establish 5q SMA carrier status in the parents. Parents of newborns with a homozygous deletion of exon 7 of the *SMN1* gene were examined to determine the number of copies of *SMN1* and *SMN2* with quantitative MLPA using the SALSA MLPA Probemix P060 SMA Carrier Kit (MRC Holland, Amsterdam, the Netherlands) according to the manufacturer’s protocol. The result of the *SMN2* gene copy number was given on days 7–12 of the newborns’ lives. The coding sequences and adjacent intronic regions of exons 7 and 8 of the *SMN1* gene (NM_00344.3) (exon 7—5′GTGAAACAAAATGCTTTTTAACATCC, 3′CCATAAAGTTTTACAAAAGTAAGATTCAC, exon 8—5′GGTTTAACTGGAATTCGTCAAGC, 3′CAAATTTTCTCAACTGCCTCACC) were analyzed via direct automated Sanger sequencing for the identification of exon 7 of *SMN2* splicing-modifier variants.

Upon completion of the diagnostic testing, parents were immediately summoned to the Russian Children Neuromuscular Center of the Veltischev Clinical Pediatric Research Institute of Pirogov Russian National Research Medical University for an appointment with a specialist for consultation. In the case of a 5q SMA diagnosis confirmation, the doctor could prescribe treatment with medications already registered or available in the Russian Federation within early access programs or recommend inclusion into clinical studies approved by the Russian Healthcare Ministry if they met the inclusion criteria.

## 3. Results

The samples were brought by courier every day to the Research Center for Medical Genetics, where each sample was also recorded. Newborn screening at the Research Center for Medical Genetics was performed twice weekly by melting curve analysis using the SALSA^®^ MC002 SMA Newborn Screen Kit (MRC Holland). Each sample was analyzed by the melt assay within 3 working days according to the screening protocol. Cases with clear positive or ambiguous results were immediately referred for confirmatory testing by PCR-RFLP analysis, which was completed within 3 days according to the laboratory protocol. In fact, all three true positives in this study were confirmed within five days after receipt of the original DBS sample.

In this pilot project, of the 23,405 neonates that were screened, three were confirmed to have homozygous deletions of exon 7 of the *SMN1* gene. The prevalence of 5q SMA was 1:7801. The following quantitative MLPA analysis detected zero copies of *SMN1* and two copies of *SMN2* in one patient and zero copies of *SMN1* and three copies of *SMN2* in two other patients.

Family analysis was carried out for all newborns with homozygous deletions of exon 7 of the *SMN1* gene. Both parents were confirmed to carry the heterozygous deletion of exon 7 of the *SMN1* gene in all three families. The parents of the first newborn with a positive result and his sibling had one copy of *SMN1* and one copy of *SMN2* each. The mother of the second newborn had one copy of *SMN1* and one copy of *SMN2*, and the father had one copy of *SMN1* and three copies of *SMN2*. The parents of the third newborn had one copy of *SMN1* and two copies of *SMN2* each; the older sibling had zero copies of *SMN1* and three copies of *SMN2*; and the second sibling had two copies of *SMN1* and two copies of *SMN2*. It is worth noting that in one of the families, the older sibling also has 5q SMA, which was unknown at the time of obtaining the screening results. The neonate’s mother was accidentally hospitalized in a maternity ward where newborn screening for 5q SMA was taking place. In Moscow, pregnant women are admitted to maternity wards by ambulance, depending on the number of maternity beds and available delivery rooms, the distance to the pregnant woman’s place of residence, and her labor activity. Additionally, information about the pilot project has not been advertised anywhere. The mother of an older child affected by 5q SMA was admitted by ambulance to the maternity ward, where she participated in the pilot project. The proband’s sibling has type II 5q SMA and receives Nusinersen treatment.

Aside from the deletion of exon 7 of the *SMN1* gene, the SALSA^®^ MC002 SMA Newborn Screen Kit (MRC Holland) allows for the detection of homozygous deletion of exon 7 of the *SMN2* gene. This deletion was detected in 1338 newborns. The frequency of this deletion in a homozygous state, according to the results of the pilot screening, was 1:18, or 0.056.

During the organization of the pilot screening project, we were faced with the following problems in the preanalytical stage of diagnostics: (1) an inconsistency between the number of samples of newborns in the database and the number of forms delivered to the laboratory; and (2) a problem with summoning families for validation of positive results related to the depersonalization of the newborns’ samples. The first problem was solved by constantly checking the numbers of test forms from maternity wards and registering them at the Research Center for Medical Genetics, as well as checking the numbers inside the laboratory and registry of the Research Center for Medical Genetics. For full population screening, a barcode system should be introduced to minimize human error. The second problem was more difficult; the maternity ward was not always quick to respond when providing information about a positive screening result. The rapid recall of parents and their newborn babies was achieved thanks to the high quality of both the specialists at the Research Center for Medical Genetics registry office and the co-authors of the article. The acquired experience was taken into account when preparing the mass newborn 5q SMA screening program in the Russian Federation.

**Figure 2:**
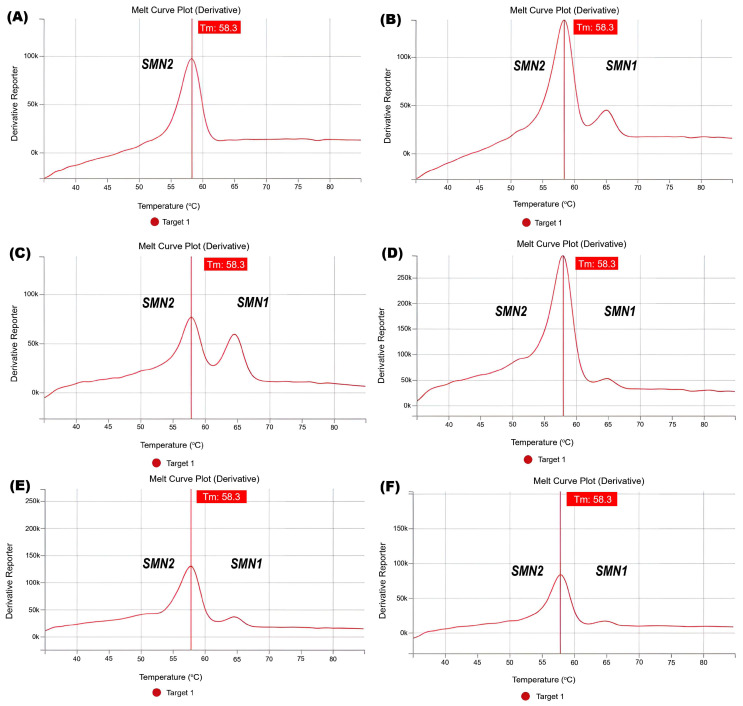
Cases with complicated interpretation detected during the newborn screening. Melt curves from the SALSA NBS assay are shown. Please see the text for a detailed explanation of the results shown in panels (**A**–**F**).

During the pilot, we interpreted 219 of 23,405 (0.9%) results as “ambiguous”, for the following reason. Despite the fact that there was a clear signal in the temperature range corresponding to exon 7 of the *SMN1* gene, its intensity was significantly (5–9 times) lower than the intensity of the signal of exon 7 of the *SMN2* gene, while during the analysis of other samples the intensity ratio did not exceed 1:2–1:3 (Figure 2). Because the SALSA^®^ MC002 SMA Newborn Screen Kit (MRC Holland) includes a control sample with an intensity ratio of 1:5, we selected all the samples with lower ratios for the PCR-RFLP assay using DNA extracted from the DBS. All such samples were analyzed with PCR-RFLP using DNA extracted from dried blood spots. All these 219 ambiguous cases were analyzed with PCR-RFLP using DNA extracted from the DBS. We were able to obtain the blood samples of five newborns from the group of 219 ambiguous cases and perform MLPA analysis on them from their blood samples. Five available samples of the newborns were analyzed with quantitative MLPA using DNA extracted from blood. In these cases, one copy of the *SMN1* gene and three or four copies of the *SMN2* gene were detected. These additional tests were carried out on samples with ambiguous results, which were not part of the screening program. Thus, we confirmed that the abnormal peak ratios in these “ambiguous” cases were caused by the multifold prevalence of *SMN2* copies over *SMN1* in the examined samples. These results should therefore not be regarded as false positives.

In the SALSA MC002 SMA Newborn Screen, PCR amplification of exon 7 of the *SMN1* gene and the closely related *SMN2* gene is performed, followed by fluorescent probe binding to the amplicons and generation of a melt curve. Fluorescence is only measured during melt curve generation. Examples of the screening assay results from controls and ambiguous cases are shown in Figure 2. Red indicates the sample that we analyzed. In the sample in which the signal from exon 7 of the *SMN2* gene was registered, the signal from exon 7 of the *SMN1* gene was not registered—the control of deletion of exon 7 of the *SMN1* gene (from SALSA^®^ MC002) (Figure 2A). The sample in which the signal from exon 7 of the *SMN1* gene and the signal from exon 7 of the *SMN2* gene were registered in the intensity ratio 1:5 (the normal control from SALSA^®^ MC002) (Figure 2B). The sample that showed a signal from exon 7 of *SMN1* and *SMN2*, a control of normal genotype that was then validated by MLPA (Figure 2C). Samples that showed an intensity ratio of approximately 1:9 higher than the control in the SALSA^®^ MC002 (Figure 2D–F). However, a signal from exon 7 of *SMN1* and *SMN2* is detected. These samples were validated by PCR-RFLP. These samples were also analyzed with quantitative MLPA. According to the MLPA results for these samples, the following genotypes are shown: one copy of *SMN1* and three copies of *SMN2* per genome (Figure 2D); one copy of *SMN1* and four copies of *SMN2* per genome (Figure 2E); and one copy of *SMN1* and three copies of *SMN2* per genome (Figure 2F).

As of November 2022, there have been no reported cases of 5q SMA in newborns that screened negative in the pilot study, indicating that the sensitivity of the NBS test was 100% in this small population. When the NBS test is extended to larger populations, the sensitivity would be expected to fall to 95–98% owing to the method’s inability to detect pathogenic mutations other than homozygous exon 7 deletions.

## 4. Discussion

5q SMA is one of the most severe neuromuscular disorders and the most common genetic cause of child mortality and disability. The disease is characterized by progressive symptoms of wasting palsy due to the degeneration of alpha-motor neurons in the anterior horns of the spinal cord. Newborn screening for 5q SMA allows the identification of presymptomatic patients and the prescription of gene therapy. Newborn screening for 5q SMA has been carried out in different countries by different methods (Table 1).

The pilot newborn screening project was conducted for the first time in the Russian Federation from August 2019 to January 2022. On 1 June 2021, the Premier Minister announced the expansion of the national newborn screening program from the current five to thirty-six diseases, including 5q SMA, which is expected to start in 2023 in all regions of the Russian Federation. In 2022, pilot projects have been implemented in six regions of the Russian Federation, with up to 200,000 newborns screened during the year. Screening for 5q SMA is implemented by the qPCR method. In St. Petersburg, the active pilot program started in October 2021. NBS 5q SMA is currently being carried out in five hospitals across St. Petersburg, and so far, no cases of 5q SMA have been identified. This pilot has also enabled researchers to identify carriers (five carriers have been identified to date). Preparations for the all-country national program are now ongoing, with structure/pathways established and protocols reviewed for approval [29].

According to the results of the pilot newborn screening project for 5q SMA in Moscow, the prevalence of the disease was 1 in 7801 neonates. The estimated prevalence of 5q SMA in the Moscow region is 1 in 5184 [3,4,5].

The observed 5q SMA prevalence in Moscow is lower than expected, although there is no significant difference between these figures (χ^2^ = 0.774, *p* = 0.771). The same situation can be observed in Germany, the USA, Taiwan, Australia, and Canada [13,14,17,20,23]. In all these countries, the observed 5q SMA prevalence is lower than the estimated prevalence, calculated from the carrier frequency for the deletion of exon 7 of the *SMN1* gene, although it stays within the confidence interval for the estimated prevalence. For some countries, the difference can be caused by the small cohort sizes (approximately 100 people in Taiwan, Australia, and Canada) used to calculate the 5q SMA carrier frequency. Aside from that, some authors mention other causes for the difference between estimated and expected disease prevalence: (1) a lethal genotype—0 copies of the *SMN1* gene and 0 copies of the *SMN2* pseudogene (in this case, the embryo development stops at early terms of pregnancy), and (2) awareness of future parents about their carrier status with the conduct of prenatal and pre-implantation diagnostics. The data on the expected 5q SMA prevalence in Latvia was obtained according to the 10 year analysis of 5q SMA incidence, calculated per number of births during this period. This data cannot be compared with the data from other countries because some neonates with 0-I 5q SMA could die before receiving a diagnosis, and patients with type IV could have no symptoms at the moment of analysis. On the other hand, in Italy, the estimated and expected prevalence of 5q SMA correspond almost exactly.

In China, during the newborn screening, more cases of homozygous deletion of exon 7 of the *SMN1* gene were detected than were estimated according to the calculations, although the observed prevalence is, similar to the results in other countries, within the confidence interval of the expected prevalence. This can possibly be connected to the bias in the population cohort in which the carrier frequency was calculated: it consisted of pregnant women from various regions of the People’s Republic of China [27].

In Japan, during the examination of more than 22,000 newborns, no homozygous carriers of the deletion of exon 7 of the *SMN1* gene were detected. No published data on 5q SMA carrier frequency in this country was available; however, considering the incidence rate of 5q SMA (3.09 per 100,000 live births), which is by an order of magnitude lower than in other countries [5,14,16,17,21,29], this result is not unexpected.

## 5. Conclusions

At the moment, there are three kinds of medication registered in the Russian Federation for 5q SMA treatment. The goal of the newborn screening is the early detection of patients with 5q SMA for presymptomatic treatment. The rapid diagnosis in the presymptomatic stage of 5q SMA and the early referral to gene therapy for the treatment of newborns with a positive result during the pilot newborn screening project in Moscow were possible mostly due to the fact that the project was based and carried out within one city.

The prevalence of detected 5q SMA cases does not have significant differences from the estimated prevalence; however, similar to the results in other countries, there is a noticeable tendency towards a decrease in the observed prevalence compared to the expected prevalence. Our data is within the confidence interval, although the incidence rate is lower. The previously estimated incidence rate was based on an indirect method, through the incidence rate in the Russian population. We present the incidence rate estimated by the direct method.

The pilot 5q SMA screening was the first mass screening in the Russian Federation to use DNA diagnostic methods. The experience acquired during its implementation became the basis for planning a 5q SMA screening covering the entirety of the Russian Federation. The aim of the pilot project for newborn screening for SMA was not to study its economic efficiency. We studied the need for screening newborns for SMA in the Russian Federation since three drugs have been registered in the country at the moment. We have studied the advantages of the method, faced with ambiguous samples and various issues in the organization, and how the screening was conducted. The data will be taken into account when implementing expanded screening for 32 diseases in the Russian Federation in 2023. Our experience allowed us to organize another pilot project in 2022, which will include newborns from six regions of the Russian Federation.

In summary, this project has demonstrated the possibility, reality, and necessity of newborn screening for 5q SMA in the Russian Federation. The newborn screening showed data similar to the results in Europe. The pilot newborn 5q SMA screening project formed the basis for the implementation of 5q SMA newborn screening for the entire country. However, mass newborn screening for the entire country of Russia will be implemented using a different method for screening for SCID/5q SMA.

## Figures and Tables

**Table 1:** Data on newborn screening in an array of countries, including the new 5q SMA pilot screening in Russia. qPCR: quantitative Polymerase Chain Reaction; ddPCR: droplet digital Polymerase Chain Reaction; MLPA: multiplex ligation-dependent probe amplification; var methods: various methods.

Country/Region	Project Start Month	Carrier Frequency/Estimated 5q SMA Prevalence	Methodology Tier 1/Tier 2	Detected Cases per Number of Newborns	5q SMA Prevalence According to the Screening Results	Estimated 5q SMA Prevalence
Taiwan	11/14	1:48 [11]	qPCR/ MLPA/	7:120,267 [12]	1:17,181 [13]	1:8968
USA	01/16	1:53 [14]	var methods ddPCR-qPCR	180:2,395,718 [13,15]	1:13,310 [1]	1:11,236
Germany	01/18	1:30 [16,17]	qPCR/ MLPA	43:297,163 [18]	1:6911 [13]	1:3600
Belgium	03/18	No data	qPCR/ MLPA	9:136,339 [13,19]	1:15,149	No data
Australia	08/18	1:49 [20]	qPCR/ ddPCR	18:202,388 [13]	1:11,244	1:6724
Italy	09/19	1:35 [21]	qPCR/ qPCR	15:90,885 [22]	1:6059	1:4900
Canada	01/20	1:54 [23]	Mass/ MLPA	5:139,810 [13,24]	1:27,962	1:11,664
Japan	05/20	1:32,362 (according to the retrospective analysis data 2007–2016) [25]	qPCR/ MLPA	0:22,209 [13]	Not detected	1:32,362
Japan (Osaka)	02/21	1:32,362	qPCR/ MLPA	0:10,000 [26]	Not detected	1:32,362
China	03/18	1:53 [27]	The Agena iPLEX assay (Mass)/ MLPA	3:29,364 [28]	1:9788	1:11,236
Latvia	02/21	1:9091 (according to the retrospective analysis data 2007–2017) [29]	qPCR/ qPCR and MLPA	2:10,411 [30]	1:5206	1:9069
Russia (Moscow)	08/19	1:36 1:5184 [3]	The Melt Assay/ MLPA	3:23,405	1:7801	1:5184

**Figure A1:**
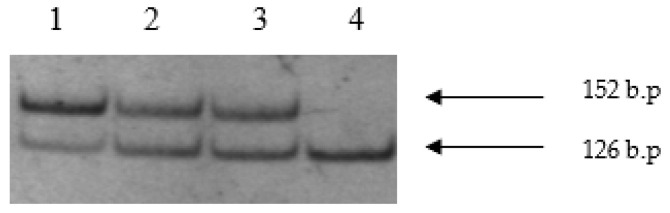
Analysis of the exon 7 deletion of the *SMN1* gene. A deletion is registered in the absence of a band of the appropriate length (track No. 4). Figure A1 illustrates fragments of electropherograms with analyses of DNA samples from a patient with 5q SMA (lane 4), his parents (lanes 2 and 3), and a healthy control (lane 1).

**Figure A2:**
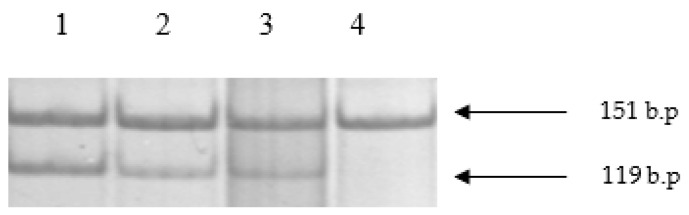
Analysis of the deletion of exon 8 of the *SMN1* gene. A deletion is registered in the absence of a band of the appropriate length (track No. 4). Figure A2 illustrates fragments of electropherograms with analyses of DNA samples from a patient with 5q SMA (lane 4), his parents (lanes 2 and 3), and a healthy control (lane 1).

## Data Availability

Not applicable.

## Appendix A

The PCR-RFLP method allows sequences of the *SMN1* gene and its homologue, *SMN2*, to be separated by restriction fragment length polymorphism analysis and to determine whether a deletion in exons 7 and/or 8 is the cause of 5q SMA (Table A1).

**Table A1:** Composition of PCR Mix SMA-7-8.

Number of Tubes	PCR Buffer, µL	dNTP, µL	Primer (7 or 8) Mix Volume, µL	The Volume of Taq Polymerase, µL	Volume of H_2_O, µL
1	6	2.5	0.7	0.2	13.6

PCR program for exon 7: hot start 95°—3 min

(1) $$32\;\mathrm{cycles}\;\;\;\;\;\left\{\begin{array}{c} 94^{{}^{\circ}}—45\;\sec\\ 55^{{}^{\circ}}—45\;\sec\\ 72^{{}^{\circ}}—45\;\sec \end{array}\right.$$

final elongation: 72°—7 min

PCR program for exon 7: hot start 95°—3 min

(2) $$28\;\mathrm{cycles}\;\;\;\;\;\left\{\begin{array}{c} 94^{{}^{\circ}}—45\;\sec\\ 57^{{}^{\circ}}—45\;\sec\\ 72^{{}^{\circ}}—45\;\sec \end{array}\right.$$

final elongation: 72°—7 min

Perform the restriction assay:

(1) Prepare 0.5 mL restriction tubes by numbering and arranging them appropriately in a rack. Add 10 µL of amplificant from the corresponding tube to each tube after PCR;
(2) Prepare a restriction mixture consisting of buffer, enzyme, and water according to Table A2.

**Table A2:** The restriction mixture for exon 7 restriction (enzyme Bse 8I (Thermo Scientific)) and for exon 8 restriction (enzyme Bse 1I (Thermo Scientific)).

Number of Tubes	Volume of 10X Restriction Buffer, µL	Volume of H_2_O, µL	Enzyme Volume, µL
	1.5	2.9	0.6

Mix the mixture thoroughly and add 5 µL to each tube with the post-PCR sample. Close the lid, centrifuge for 2–3 s and then incubate at 65 °C for 3 h (exon 8, enzyme Bse 1I) or 4 h at 60 °C (exon 7, enzyme Bse 8I).

### Appendix A.1. Analysis of Results

The fragments are analyzed in a 7% acrylamide gel (29:1 AA/BA stock solution). Electrophoretic separation of the fragments is carried out at a field strength of 16 V/cm for 0.5–1.0 h (by the end of fragment separation, the leading dye bromophenol blue should have passed a distance of two thirds of the gel length).

The length of the amplified fragment of exon 7 is 152 bp, and exon 8 is 188 bp. After the restriction is carried out, the following size bands are registered on the gel (Figure A1 and Figure A2):

exon 7 of the *SMN1* gene—152 b.p. exon 8 of the *SMN2* gene—151 b.p.
exon 7 of the *SMN2* gene—126 b.p. exon 8 of the *SMN1* gene—119 b.p.
